# Post-Vaccination SARS-CoV-2 Infections among Health Workers at the University Hospital of Verona, Italy: A Retrospective Cohort Survey

**DOI:** 10.3390/vaccines10020272

**Published:** 2022-02-10

**Authors:** Stefano Porru, Gianluca Spiteri, Maria Grazia Lourdes Monaco, Alessandro Valotti, Angela Carta, Virginia Lotti, Erica Diani, Giuseppe Lippi, Davide Gibellini, Giuseppe Verlato

**Affiliations:** 1 Section of Occupational Medicine, Department of Diagnostics and Public Health, University of Verona, 37134 Verona, Italy; stefano.porru@univr.it (S.P.); angela.carta@univr.it (A.C.); 2 Clinical Unit of Occupational Medicine, University Hospital of Verona, 37134 Verona, Italy; gianluca.spiteri@aovr.veneto.it; 3 Postgraduate School of Occupational Medicine, University of Verona, 37134 Verona, Italy; alessandro.valotti@studenti.univr.it; 4 Section of Microbiology, Department of Diagnostics and Public Health, University of Verona, 37134 Verona, Italy; virginia.lotti@univr.it (V.L.); erica.diani@univr.it (E.D.); davide.gibellini@univr.it (D.G.); 5 Section of Clinical Biochemistry, University of Verona, 37134 Verona, Italy; giuseppe.lippi@univr.it; 6 Unit of Microbiology and Virology, University Hospital of Verona, 37134 Verona, Italy; 7 Unit of Epidemiology and Medical Statistics, Department of Diagnostics and Public Health, University of Verona, 37134 Verona, Italy; giuseppe.verlato@univr.it

**Keywords:** COVID-19 pandemic, SARS-CoV-2 vaccination, health workers, health surveillance, infection spreading, COVID-19 symptoms

## Abstract

Background: The SARS-CoV-2 vaccination campaign began on 27 December 2020 in Europe, primarily involving health workers. This study aimed to assess the SARS-CoV-2 vaccination effectiveness, as assessed by reductions in incidence, symptom severity, and further infection spreading. Methods: A retrospective cohort study was conducted on 9811 health workers operating at the Verona University Hospital, Italy, from 27 December 2020 to 3 May 2021. All health workers were offered vaccination with Comirnaty (BNT162b2, BioNTech/Pfizer, Mainz, Germany/New York, United States), and a health surveillance program was implemented with periodical swab testing. Vaccination status and clinical data were collected using an ad hoc semi-structured questionnaire and health surveillance charts. Results: As of 3rd of May, 82.5% of health workers had been vaccinated against SAR-CoV-2, and 177 (1.8%) had tested positive for SARS-CoV-2. Vaccination more than halved the cumulative incidence of SARS-CoV-2 infection and reduced by two-thirds the cumulative incidence of symptomatic subjects. In detail, most unvaccinated HWs were symptomatic; 50% reported fever, 45% reported ageusia/anosmia, and nearly 20% reported dyspnea. These percentages were much lower in HWs who had been vaccinated for at least 14 days (18% for fever and anosmia, 6% for dyspnea and ageusia). Moreover, cases of vaccine breakthrough were sixfold less likely to further spread the infection than unvaccinated HWs. Conclusions: SARS-CoV-2 vaccination reduced the infection frequency among HWs, further spreading of the infection, and the presence, severity, and duration of COVID-19-related symptoms.

## 1. Introduction

Since the onset of SARS-CoV-2 in December 2019 in Wuhan (China), many countries have experienced multiple waves of outbreaks of this virus. As of 16 January 2022, almost 319 million cases have been diagnosed worldwide; over 5,500,000 were fatal [1,2]. Italy was one of the first affected countries, with more than 5 million confirmed cases and about 138,000 deaths caused by the infection [3,4]. Health workers (HWs) are the category most exposed to infection; since February 2020, about 150,000 cases have been diagnosed among Italian health workers, with over 9000 hospital admissions [4].

Since the pandemic’s beginning, an enormous effort to reduce the pandemic’s impact has involved the entire scientific community. Until 2020, containment measures against SARS-CoV-2 diffusion mostly included hand hygiene, respiratory hygiene, self-isolation, and social distancing. At the end of 2020, the first vaccine was already made available, and as of December 27th 2020, many SARS-CoV-2 vaccination campaigns had begun, especially in developed countries [5]. Priority in vaccine distribution was given to the populations at higher risk of exposure, such as HWs, who were both victims and spreaders of the virus. In this context, protecting HWs from COVID-19 is also pivotal in preserving and safeguarding healthcare systems [6]. 

As of 16 January 2022, more than 8 billion doses of the vaccine have been administered worldwide, including first and second doses [2]. More than 88% of the population over the age of 12 has already received at least one dose of vaccine in Italy, and 96% of the vaccinated subjects have completed the vaccination course [7]. Despite the good vaccine coverage (consistent with that in other European countries), specific segments of the population remain hesitant toward the vaccine even in the presence of chronic conditions [8].

The vaccination efficacy and effectiveness represent critical points in the fight against the pandemic [9]. The outcome of SARS-CoV-2 infection in individuals is heterogeneous and related to several variables, including age, sex, and pre-existing morbidity, so specific endpoints must be defined to evaluate it properly, and all determinants should be considered [10].

The transmission dynamics of SARS-CoV-2 represent a relevant aspect, particularly regarding what proportion of coronavirus disease 2019 (COVID-19) spread is associated with the transmission of infection from presymptomatic or asymptomatic persons [11]. Several population clusters where patients acquired SARS-CoV-2 from infected sources without symptoms have been described [12], supporting the role of multiple measures of control, such as mask-wearing, social distancing, and strategic testing to control SARS-CoV-2 diffusion, in addition to vaccine administration [11]. Moreover, the power of a vaccine to protect against severe disease and mortality is a crucial endpoint. Data on vaccination effectiveness in real-world settings, assessing the reduction in SARS-CoV-2 infections, hospitalizations, and deaths caused by COVID-19, are now available [13]. 

Another aspect of this pandemic regards both reinfections and infections among vaccinated subjects. While, on the one hand, the pandemic growth curve has been significantly reduced, on the other hand, in various health contexts worldwide, cases of reinfection have been detected in subjects previously positive for SARS-CoV-2, along with cases of infection diagnosed in vaccinated subjects (conventionally called “vaccine breakthrough”) [14,15,16,17]. 

This issue is also related to SARS-CoV-2 variants spreading worldwide, with likely increased transmissibility and virulence and possibly lesser vaccine protection response. According to Fiolet et al. [18], overall, the efficacy of vaccines could be reduced for Delta infection, but they still offer a high level of protection against severe COVID-19 and hospitalization for all variants after full immunization. However, on this aspect, the available research is not entirely consistent. The reaction of vaccinated and unvaccinated subjects towards virus variants differs across the several studies conducted in different parts of the world [19]. Lastly, on November 26, 2021, the World Health Organization announced a new SARS-CoV-2 variant, Omicron (B.1.1.529); this the most mutated SARS-CoV-2 variant so far, characterized by rapid spread in the community with higher levels of incidence than previously seen in this pandemic but a lower risk of severe disease and death [20,21,22]. 

As reported above, health workers are a high-risk population for SARS-CoV-2 infection, and they were among the first to be vaccinated. For this reason, several studies on the effectiveness of vaccinations in this group of workers are becoming available. Two studies conducted on health workers in Israeli hospitals, where the vaccination campaign began a week earlier than in Italy, showed a significant decrease in the incidence of new cases among vaccinated subjects. The reduction was evident as early as the second week after the first dose. The protection was maintained throughout the 8-week observation period [23,24]. A study carried out in an Italian healthcare context showed that healthcare workers who had completed the full vaccination course were at lower risk of infection (by 2.4-fold) than those vaccinated with only one dose or none [25]. Bergwerk et al. reported that among fully vaccinated health care workers, the rate of SARS-CoV-2 breakthrough was correlated with neutralizing antibody titers, and most of them were mild or asymptomatic, although persistent symptoms did occur [26]. According to Alishaq et al., the presence of symptoms and contact with confirmed cases lead to a higher risk of infection after vaccination among healthcare workers; this shows that screening, even after full vaccination, should prioritize these groups. The authors highlighted that comorbidities were not associated with a higher risk of breakthrough infection in their research. In comparison, multiple comorbidities have been reported to be associated with a higher risk of SARS-CoV-2 infection among unvaccinated persons. The reasons for this disagreement are still unclear [27]. In an effectiveness study, Chodick et al. found that the BNT162b2 mRNA vaccine was associated with a 51% reduced risk of SARS-CoV-2 infection, with 54% vaccine effectiveness against symptomatic infection from 13 to 24 days after immunization with the first dose of BNT162b2 [28]. Scobie et al. reported the incidence rate ratio for cases among subjects who were not fully vaccinated, compared with those among fully vaccinated persons; the ratio decreased from 11.1 to 4.6 between periods where the Delta variant’s prevalence was low to high [29].

The present study aimed at investigating the association between SARS-CoV-2 vaccination status and SARS-CoV-2 infection incidence, spread, and COVID-19 symptom severity among HWs of a large university hospital in the Veneto Region, Italy.

## 2. Subjects and Methods

### 2.1. Study Design, Setting, and Population

A retrospective cohort study was conducted within an HW surveillance project framework to assess SARS-CoV-2 vaccination effectiveness among 9811 HWs operating at the University Hospital of Verona, Italy [30,31], one of Italy’s largest hospitals in terms of bed number (about 1500 beds).

Enrollment started on 27 December 2020 and was completed on 3 May 2021. Each subject was followed up to 3 May or the first positive RT-PCR result.

For data analysis, the working population was grouped into three categories based on job tasks, i.e., administrative, physician (including resident), or nurse and other health professionals (also including medical students and students of medical-related professions).

### 2.2. Vaccine Administration

All HWs were offered vaccination with Comirnaty (BNT162b2, BioNTech/Pfizer, Mainz, Germany/New York, NY, United States), after an assessment of possible contraindications and precautions. The dosing schedule for the BNT162b2 vaccine was a 2-dose series administered 21 days apart [32]. All vaccine recipients were observed for 15–30 min to manage immediate adverse reactions promptly [33]. Vaccination status was ascertained from the employee’s health database. 

HWs could pass through three different statuses during the follow-up, according to vaccination history: “Group A” if they had not received any dose of vaccine yet (unvaccinated) or had been administered the 1st dose less than 14 days prior; “Group B” from the 14th day after the 1st dose until the 13th day after the 2nd dose; and “Group C” (fully vaccinated) from the 14th day after the 2nd dose to 3rd May 2020 [34,35]. Hence, an HW receiving the two doses of vaccine passed through all three statuses, while an HW refusing vaccination remained in Group A.

### 2.3. SARS-CoV-2 Health Surveillance 

According to national and loco-regional regulations [36], health surveillance through RT-PCR testing was implemented every 8 days or every 20 days based on the exposure risk definition and regardless of vaccination status. In particular, the risk of exposure to SARS-CoV-2 was classified as high (for persons working in the emergency department, dedicated COVID-19 units, and all units caring for fragile patients) or low (for other population subgroups). This approach enabled monitoring of the spread of the infection, even among vaccinated subjects. Although the test was not mandatory, acceptance was over 98%, regardless of vaccination status.

### 2.4. Symptom Definition and Data Collection of Clinical Infection

The positive subjects (HWs with at least one positive RT-PCR test) were interviewed using an ad hoc semi-structured questionnaire to investigate: adverse drug reactions, possible reinfection, presence or absence of symptoms, any contact with positive cases post-vaccination, and spreading.

Participants infected by SARS-CoV-2 were defined as symptomatic if they had any of the following: temperature greater than 37.6 °C (fever), headache, sore throat, cough, dyspnoea, rhinorrhoea, myalgia/malaise, or loss of sense of taste or smell. 

The questionnaire outline is available as Supplementary Material Table S1.

### 2.5. Pre-Existing Medical Conditions 

For 163 positive HWs (92%), the health surveillance chart review acquired baseline clinical status with occupational physician support. The following pre-existing medical conditions (PMCs) were considered: cardiovascular diseases (e.g., hypertension, cardiac arrhythmia, cardiac ischemia), respiratory diseases, allergies, diabetes, neoplasms diagnosed in the last five years, autoimmune diseases, haematological diseases, neurological diseases, and acquired immunosuppression.

### 2.6. Outcomes and Endpoints 

The primary outcome included vaccine effectiveness, investigated by examining the SARS-CoV-2 infection incidence and its spreading and symptoms.

The incidence of infection was calculated considering new positive SARS-CoV-2 infection cases (as above defined) diagnosed from 27 December 2020 to 3 May 2021, distinguishing Group A, Group B, and Group C, as above described.

Disease severity was evaluated based on symptom presence or absence (symptomatic, asymptomatic), their duration (1–3 days, 3–7 days, >7 days), and need of hospitalization. The influence of vaccination status and PMCs on the outcomes of disease severity was also evaluated. For this purpose, these diseases were coded as (0) absent; (1) one PMC; (2) two PMCs; or (3) three or more PMCs.

According to the rules issued by the national health authorities regarding the epidemiological surveys to be conducted in infected persons [37,38], spreading was here defined as a dichotomic variable based on the presence or absence of persons presumably infected by the enrolled subjects. Even for these people, the severity of infection was evaluated by classifying positive subjects according to the presence or absence of symptoms and need for hospitalization. 

### 2.7. Statistical Analysis

The Clopper–Pearson method was used to calculate the 95% confidence interval (CI) of cumulative incidence of SARS-CoV-2 infection. The significance of differences among groups was evaluated by Fisher’s exact test or chi-square test for categorical variables and by one-way ANOVA or Kruskal–Wallis test for quantitative variables, as appropriate. The cumulative incidence of SARS-CoV-2 infection over time was estimated by the Kaplan–Meier method as a function of vaccination status.

As a sensitivity analysis, those who completed 2 doses and those who remained unvaccinated were compared by plotting cumulative incidence from the 1st of March until the 3rd of May, using the Kaplan–Meier method. The second half of the observation period was chosen, as the incidence in the Verona area was more stable then than in January and February. The significance of differences was evaluated by the log-rank test, and multivariable survival analysis was accomplished using the Cox model. The proportional hazard assumption of the Cox model was tested by graphical methods. The analyses were performed in Stata 14 (StataCorp, College Station, TX, USA), and statistical significance was set at *p* < 0.05.

## 3. Results

As of 3 May 2021, within a total population of 9811 HWs, 8093 were vaccinated against SARS-CoV-2 (82.5%).

Table 1 shows the demographic and occupational characteristics of HWs enrolled by SARS-CoV-2 vaccination status.

The incidence of SARS-CoV-2 infection among HWs of the University Hospital of Verona was 101.4 per 100,000 per week (95% CI 87.5–117.5) during the observation period. Incidence was the highest among the HWs in Group A (147.2/100,000/week, 95% CI 123.5–175.4), intermediate among those in Group B (69.8/100,000/week, 44.0–110.8), and the lowest among Group C (51.6/100,000/week, 95% CI 36.8–72.2).

**Figure 1:**
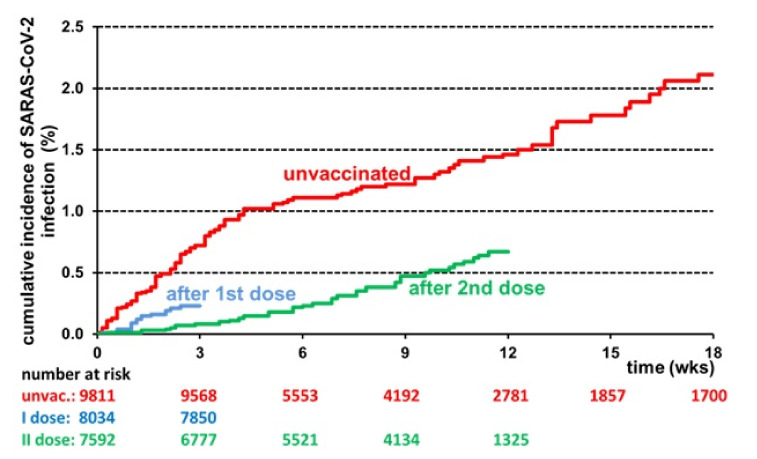
Cumulative incidence of SARS-CoV-2 infections in 9811 HWs of the University Hospital of Verona, Italy, estimated by the Kaplan–Meier method. Time zero was the start of the vaccination campaign (27 December 2020) for the red curve (Group A), 14 days after the 1st dose for the blue curve (Group B), and 14 days after the 2nd dose for the green curve (Group C).

As Figure 1 shows, the cumulative incidence of SARS-CoV-2 infection was greatly reduced among Group C HWs compared to Group A HWs. The percent reduction amounted to 89% at 3 weeks after full vaccination, 79% after 6 weeks, 61% after 9 weeks, and 54% after 12 weeks. The reduction was even larger when considering symptomatic COVID-19. No symptomatic infection was recorded in the first 3 weeks after full vaccination, while the percent reduction accounted for 88%, 75%, and 67% after 6, 9, and 12 weeks, respectively.

During the observation period, 177 HWs (1.8%) developed SARS-CoV-2 infection, diagnosed via RT-PCR. As reported in Table 2, this subgroup had a median age significantly higher than that of the non-infected cohort (*p* < 0.001), higher prevalence of nurses, and lower prevalence of physicians (*p* < 0.001). Most cases had a normal body weight (*n* = 95, 59%) and were women (*n* = 132, 74.6%), which was consistent with the sex distribution of the surveyed population; hence, the cumulative incidence of SARS-CoV-2 did not significantly differ between men (1.5%) and women (1.9%). Moreover, 100 cases (56.5%) had been vaccinated with at least one dose.

Only 2 of the 177 infected HWs, 1 unvaccinated and 1 other who was only administered the first dose, had developed a SARS-CoV-2 infection before the vaccination campaign. Reactions to vaccination were reported by 78% (*n* = 74) of vaccinated HWs; this percentage was slightly higher after the second dose (33/37 = 89%) than after the first dose (41/58 = 71%).

Through health surveillance chart examination, 80 of 163 HWs (49%) had no previous diseases, 68 (42%) had one PMC, and 15 (9%) had at least two PMCs. Twelve cases (7.3%) of SARS-CoV-2 infection had cardiovascular disease before infection, mostly hypertension (*n* = 11, 6.75%), and three (1.8%) had diabetes. No difference was found among Group A, Group B, and Group C HWs.

**Figure 2:**
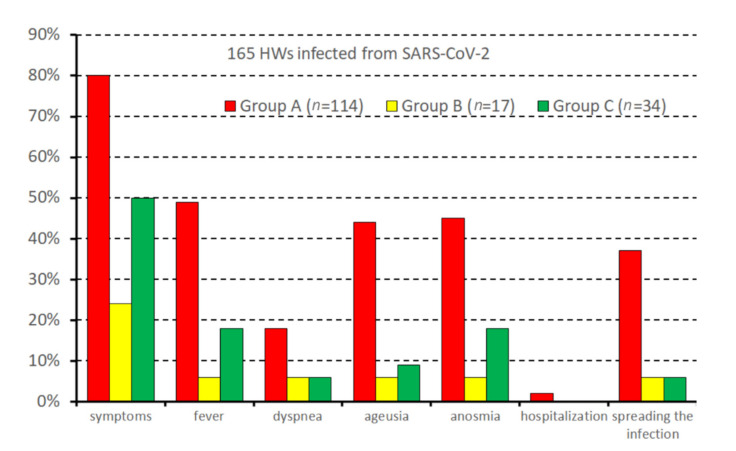
COVID-19-related symptoms, hospitalization, and infection spreading as a function of vaccination status.

Table 3 and Figure 2 show data on the infection setting, spread, and symptoms developed by 165 of 177 HWs (93%) enrolled in the study who agreed to reply to the ad hoc semi-structured questionnaire, designed for anamnestic investigation and described in Supplementary Material Table S1.

Group A also had higher risk of spreading the disease among contacts. This event was instead relatively infrequent among people who had been administered the first/second dose at least 14 days prior. Vaccination reduced by 84% the risk of disease spread. Seventy-five HWs (45%) reported having used personal protective equipment (PPE) during the contact with the source case.

The severity of COVID-19 was strongly dependent on the timing of vaccination. Most HWs among Group A were symptomatic: 50% reported fever, 45% reported ageusia/anosmia, and nearly 20% reported dyspnoea. These percentages were much lower in HWs who had been vaccinated at least 14 days prior.

The lower rate of symptoms was especially evident for fever, ageusia, and anosmia, and was also clinically relevant for the most severe symptoms, such as dyspnoea. However, this aspect did not reach the level of statistical significance. As regards hospital admission, the reduction was not significant (*p* = 1000).

Vaccination seemed also effective regarding the duration of symptoms, reducing the prevalence of all three categories (1–3 days, 4–7 days, >7 days).

### Sensitivity Analysis

Sensitivity analysis was performed on 5804 HWs who either had never been vaccinated during the study period (*n* = 1661) or were fully vaccinated with two doses at least 14 days before 28th of February 2021.

**Figure 3:**
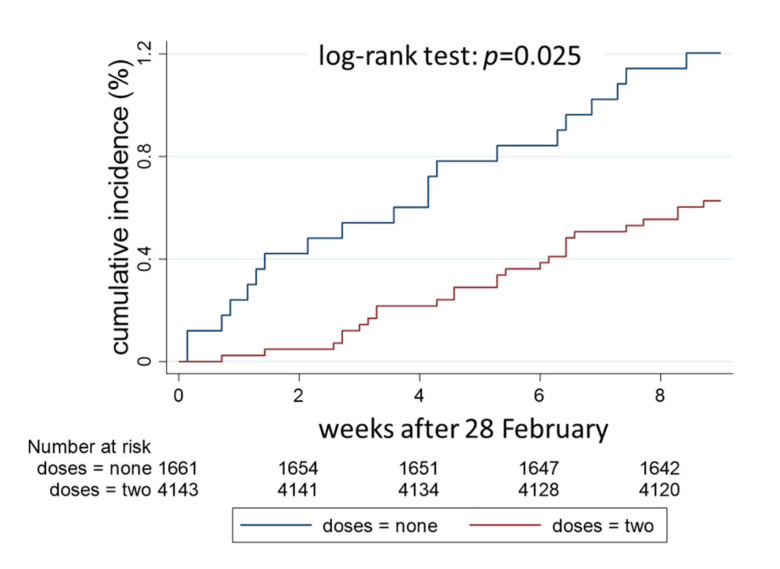
Cumulative incidence of SARS-CoV-2 infections in 5804 HWs of the University Hospital of Verona, Italy, estimated by the Kaplan–Meier method. HWs not administered any vaccine (*n* = 1661, blue line) were compared with HWs who had received two doses at least 14 days before the 28 February 2021 (*n* = 4143, red line).

The cumulative incidence of SARS-CoV-2 infection from the 1st of March until the 3rd of May was 1.20% (0.78–1.86%) in unvaccinated HWs and 0.63% (0.43–0.92%) in fully vaccinated HWs (Figure 3) (*p* = 0.025). When controlling for sex, age, and job task in a multivariable Cox regression model, the hazard ratio of SARS-CoV-2 infection in fully vaccinated HWs was 0.37 (95% CI 0.20–0.69) with respect to unvaccinated HWs (Table 4). In addition, the risk of SARS-CoV-2 infection increased with age and was not significantly affected by gender or job task.

## 4. Discussion

Nearly 4 months after the start of the vaccination campaign, high acceptance (over 80%) was recorded in our setting. Concerning this aspect, an age-related difference was highlighted: the unvaccinated group was younger than the vaccinated groups. It comprised a larger proportion of medical students and students of medical-related professions. This aspect could be attributed to the relative delayed beginning of the vaccination campaign for these groups, which started 3 months later than that for other groups (March 2021). 

The job task was also significantly associated with the acceptance of vaccination. As 3 May 2021, 22% of administrative workers were still unvaccinated, compared to nearly 17% of HWs directly involved in patient care. This difference can be explained by the progressive introduction of the mandatory vaccination, not yet existing at the beginning of the vaccination campaign. This was firstly for health workers involved in patient care [39], subsequently for other categories of workers [40], and, lastly, for the entire population aged over 50 years [41]; this last piece of legislation confirms that vaccination is a requirement for professional practice and includes access restrictions to the workplace, as well as administrative sanctions and fines.

As of 31 December 2021, about 87% of the hospital population had completed the primary vaccination course, and 83% had also received the additional booster dose.

As regarding SARS CoV-2 infections, in this study, the incidence per week among the fully vaccinated (Group C) was lower than that among the unvaccinated or recently vaccinated with one dose (Group A) cohort throughout the observation period. These data support the role of vaccination in countering the epidemic and are somewhat in line with the results of studies that investigated similar or different samples. Regarding infection transmission, Gazit et al. reported a higher percentage of effectiveness of vaccination, about 80%, compared to both unvaccinated and those vaccinated less than 7 days prior [42]. As for asymptomatic SARS-CoV-2 infection cases, more data are available on preventing the spread of infection among asymptomatic subjects, which would seem to be over 40% of all SARS-CoV-2 infections and may silently spread the virus for extended periods [43,44].

The risk of SARS-CoV-2 infection showed a growing trend in HWs directly involved in clinical activities as compared to administrative personnel, although no statistically significant differences were found among the different job tasks. This was probably due to the low number of positive cases within the groups.

The percentage of subjects infected after contact with positive HWs was significantly reduced in both Group B and Group C subjects compared to Group A (6% vs. 37%). The effectiveness concerning infection spread was as high as 84%. This percentage is higher than that reported by Shah et al., who investigated 194,362 household members of 144,525 HWs who had received at least one dose of the BioNTech–Pfizer or AstraZeneca vaccine. They showed that from the 14th post-vaccination day onwards, vaccinating a co-habiting HW was associated with a household infection rate per 100 person-years of 9.40 versus 5.93 and a hazard ratio (HR) of 0.70 (95% CI: 0.63–0.78) [45]. After the second dose, the risk of infection of household members displayed a rate per 100 person-years of 9.40 versus 2.98, with an HR of 0.46 (95% CI: 0.30–0.70). Similar results also emerged from a study by Harris et al., with 6.25% of cases among family contacts of vaccine recipients compared to 10.1% among contacts of unvaccinated HWs [46].

As regarding COVID-19 disease severity, in the examined population, there were only two cases of hospitalization; further, as reported above, vaccination status affected symptom duration. Therefore, vaccination seems to be effective in reducing symptomatic infections in the vaccinated from 14 days after the first dose. The data on symptoms are in keeping with the current literature. Hall et al. [47], in a surveillance study of HWs in the U.K. with documented baseline molecular testing and antibodies, reported that the cumulative vaccine effectiveness was 72% from 21 days after the first dose, increasing to 86% after two vaccine doses. The authors also reported a higher proportion of “classic𠇍 symptoms (63%) and 5% of asymptomatic infections among unvaccinated HWs (versus 40% with classic symptoms and 13% asymptomatic form in those who underwent vaccination). 

Different results concerning vaccination effectiveness could be biased by differences in case definition, surveillance procedures, and environmental setting. This was underlined by Fabiani et al., who estimated that, within the time intervals of 14–21 days from the first and at least 7 days after the second dose, vaccine effectiveness in preventing SARS-CoV-2 infection was 84% (95% confidence interval (CI): 40–96) and 95% (95% CI: 62–99), respectively [48].

### Strengths and Limitations

This study has some limitations to be disclosed. Data on COVID-19 symptoms were self-reported. Regarding outcomes, a limit was the assessment of spreading via an interview instead of via swab testing confirmation, as far as the household contacts are concerned. 

The sample size, especially concerning the SARS-CoV-2 spreading evaluation, seems limited as compared to other international studies. The time lag between the two doses of vaccine was short (three weeks), so the protective effect of the first dose could not be fully appraised.

As shown in Supplementary Material Figure S1, the incidence of SARS-CoV-2 infection strongly decreased at the beginning of the study period, and this time trend introduced a period effect bias. Indeed, the risk of infection tended to increase in unvaccinated HWs, who were followed up since the start of the vaccination campaign, and to decrease in vaccinated HWs, whose follow-up started two weeks after the date of vaccination. To minimize the period effect, the analysis was repeated considering the last two months of follow-up (Figure 3).

It should also be noted that although similar studies are now available, this research provides additional and thorough data, collected on almost an entire and large hospital population of HWs, well-investigated for demographic and occupational characteristics. Such data would support the overall scientific evidence, especially considering the geographical context particularly affected by the pandemic, such as Veneto in northeast Italy, a region with about 5 million inhabitants, with high employment rates and plenty of political and social initiatives with a high impact on public health.

The observation period was long enough to enable the monitoring of the effectiveness of vaccination in the medium term. The risk of infection was also assessed regarding its association with clinical data; to our knowledge, this is the only study that also reports data on students.

Finally, this study took place within a scientific framework that included complete PCR test surveillance and antibody titration follow-up, in the pre- and post-vaccination eras, of a large population of HWs. The results of the various phases of the study are published or are on the way, including data on the antibody response.

## 5. Conclusions

This study confirms that SARS-CoV-2 vaccination reduces SARS-CoV-2 infections and the symptom onset, severity, and duration of COVID-19. Vaccination also reduces infection spread in living and working environments, demonstrating again its paramount value as a preventive tool for occupational and public health.

## Figures and Tables

**Table 1:** Demographic and occupational characteristics of 9811 health workers by SARS-CoV-2 vaccination status, University Hospital of Verona, Italy.

	Group A	Group B	Group C	Total	*p*-Value
All	1718	434	7659	9811	
Sex					0.294
Male	513 (17.1)	120 (4.0)	2364 (78.9)	2997 (100.0)	
Female	1205 (17.7)	314 (4.6)	5295 (77.7)	6814 (100.0)	
Age (median, p25–p75)	31, 26–46	33.5, 26–51	37, 28–52		**<0.001**
Job task					**<0.001**
Administrative	184 **(22.2)**	32 (3.9)	612 (73.9)	828 (100.0)	
Physician	461 (16.9)	84 (3.1)	2182 **(80.0)**	2727 (100.0)	
Nurse	368 (14.9)	115 (4.7)	1980 **(80.4)**	2463 (100.0)	
Other health professionals	705 (18.6)	203 **(5.4)**	2885 (76.1)	3793 (100.0)	

*p* values were computed by Fisher’s exact test for categorical variables and by the Wilcoxon–Mann–Whitney test for continuous variables. Significant results are highlighted in bold.

**Table 2:** Main demographic and professional characteristics and vaccination status of the 9811 HWs, by SARS-CoV-2 swab test results.

	Positive (*n* = 177)	Negative (*n* = 9634)	*p*-Value
Sex			0.139
Men	45 (25.4)	2952 (30.6)	
Women	132 (74.6)	6682 (69.4)	
Age (median, p25–p75)	47, 35–55	35, 27–51	**<0.001**
Job task			**<0.001**
Administrative	12 (6.8)	816 **(8.5)**	
Physician	36 (20.3)	2691 **(27.9)**	
Nurse	70 **(39.5)**	2393 (24.8)	
Other health professional	59 (33.3)	3734 **(38.8)**	
Vaccination			**<0.001**
None	77 **(43.5)**	1641 (17.0)	
1st dose	45 **(25.4)**	389 (4.0)	
2nd dose	55 (31.1)	7604 **(78.9)**	

Categorical variables are presented as absolute frequencies (percent), while continuous variables are presented as median (p25–p75). *p* values were computed by Fisher’s exact test for categorical variables and by the Wilcoxon–Mann–Whitney test for continuous variables. Significant results are highlighted in bold.

**Table 3:** Questionnaire results referring to infection setting, symptoms, and infection spreading.

	Group A (*n* = 114)	Group B (*n* = 17)	Group C (*n* = 34)	*p* Value
Infection setting				0.064
Work	60 **(53)**	7 **(41)**	9 (26)	
Family	24 (21)	3 (18)	9 (26)	
Unknown	30 (26)	7 (41)	16 **(47)**	
Infection spreading	42 **(37)**	1 (6)	2 (6)	**<0.001**
COVID-19				
Symptoms	91 **(80)**	4 (24)	17 (50)	**<0.001**
Fever	56 **(49)**	1 (6)	6 (18)	**<0.001**
Dyspnoea	21 **(18)**	1 (6)	2 (6)	**0.126**
Ageusia	50 **(44)**	1 (6)	3 (9)	**<0.001**
Anosmia	51 **(45)**	1 (6)	6 (18)	**<0.001**
Hospitalization	2 (2)	0	0	1.000

*p* values were computed by Fisher’s exact test for categorical variables. Significant results are highlighted in bold.

**Table 4:** Influence of vaccination on the risk of SARS-CoV-2 infection in 5804 HWs, either unvaccinated during the study period (*n* = 1661) or fully vaccinated with two doses at least 14 days before 28th of February 2021. The hazard ratio and *p* values were computed via the Cox regression model, controlling for sex, age, and job task.

	Hazard Ratio (95% CI)	*p* Value
Fully vaccinated vs. unvaccinated	**0.37 (0.20–0.69)**	**0.002**
Gender (women vs. men)	0.87 (0.46–1.67)	0.682
Age (per 10-year increase)	1.61 (1.27–2.04)	**<0.001**
Job task		
Administrative	1 (reference)	
Physician	1.62 (0.43–6.11)	0.474
Nurse	2.13 (0.60–7.65)	0.245
Other health professional	2.46 (0.71–8.51)	0.154

*p* values were computed by the Wald test. Significant results are highlighted in bold.

## Data Availability

The datasets generated during the current study are not publicly available because they contain sensitive data to be treated under data protection laws and regulations. Appropriate forms of data sharing can be arranged after reasonable request to the PI.

## Supplementary Materials

The following are available online at https://www.mdpi.com/article/10.3390/vaccines10020272/s1, Table S1: Questionnaire. Figure S1: Weekly cases of SARS-CoV2 infection in the Verona Social Health Unit.
