# Supplementary material for: Post-Vaccination SARS-CoV-2 Infections among Health Workers at the University Hospital of Verona, Italy: A Retrospective Cohort Survey
Source: Vaccines (Basel). 2022 Feb 10;10(2):272. doi: 10.3390/vaccines10020272 (PMC8879605; doi:10.3390/vaccines10020272)
Supplement: Supplementary file 1 [file vaccines-10-00272-s001.zip › vaccines-1477636-supplementary.pdf]

Table S1. Questionnaire.

Topic	Questions
Positive Rt-PCR test before December 27th 2021	1. Previous SARS-CoV-2 swab positivity? □ Yes □ No
	2. If yes, please specify year and month: ……
	3. If yes, have you had any symptoms? □ Yes □ No
	4. If yes, please specify (multiple answers are possible):<br>□ rhinorrhea □ headache<br>□ headache □ malaise<br>□ cough □ dyspnea<br>□ burning or pain in throat □ ageusia<br>□ fever □ anosmia
	5. If yes, please specify duration (except for loss of sense of taste or smell):<br>□ 1-3 days □ 3-7 days □ >7 days
	6. Have you been hospitalized? □ Yes □ No
Vaccination and any adverse reaction	7. Have you been vaccinated? □ Yes □ No
	8. Did you have any side effects (adverse drug reactions) after the 1st and or after the 2nd vaccine dose?
	9. If yes, please specify (multiple answers are possible):<br>□ injection site pain □ headache<br>□ arthralgia/myalgia □ malaise<br>□ fever □ others (please specify)
	10. If yes, please specify duration:<br>□ 1-3 days □ 3-7 days □ >7 days
Symptoms associated with Rt-PCR test positive	11. Have you had any symptoms? □ Yes □ No
	12. If yes, please specify (multiple answers are possible):<br>□ rhinorrhea □ headache<br>□ headache □ malaise<br>□ cough □ dyspnea<br>□ burning or pain in throat □ ageusia<br>□ fever □ anosmia
	If yes, please specify duration (except for loss of sense of taste or smell):<br>□ 1-3 days □ 3-7 days □ >7 days
	13. Have you been hospitalized?
Contacts after December 27th 2021	14. Did you know you had contact with a SARS-CoV-2 positive person?<br>If yes, what was the setting?<br>□ Occupational □ Home □ I do not know
	15. Did the infected person show respiratory symptoms? □ Yes □ No
	16. Were you wearing personal protective equipment for the airways during contact?<br>□ Yes □ No
Contagiousness after December 27th 2021	17. Were there any people infected after contact with you? □ Yes □ No
	18. If yes, what was the setting?<br>□ Occupational □ Home □ Both
	19. If yes, how many days later the infection was diagnosed?<br>□ 1-3 days □ 3-7 days □ 8-14 days
	20. If yes, were you wearing personal protective equipment for the airways during contact?<br>□ Yes □ No
	21. If yes, has the infected person developed symptoms? □ Yes □ No
	22. If yes, has the infected person been hospitalized due to Covid-19 related symptoms? □ Yes □ No

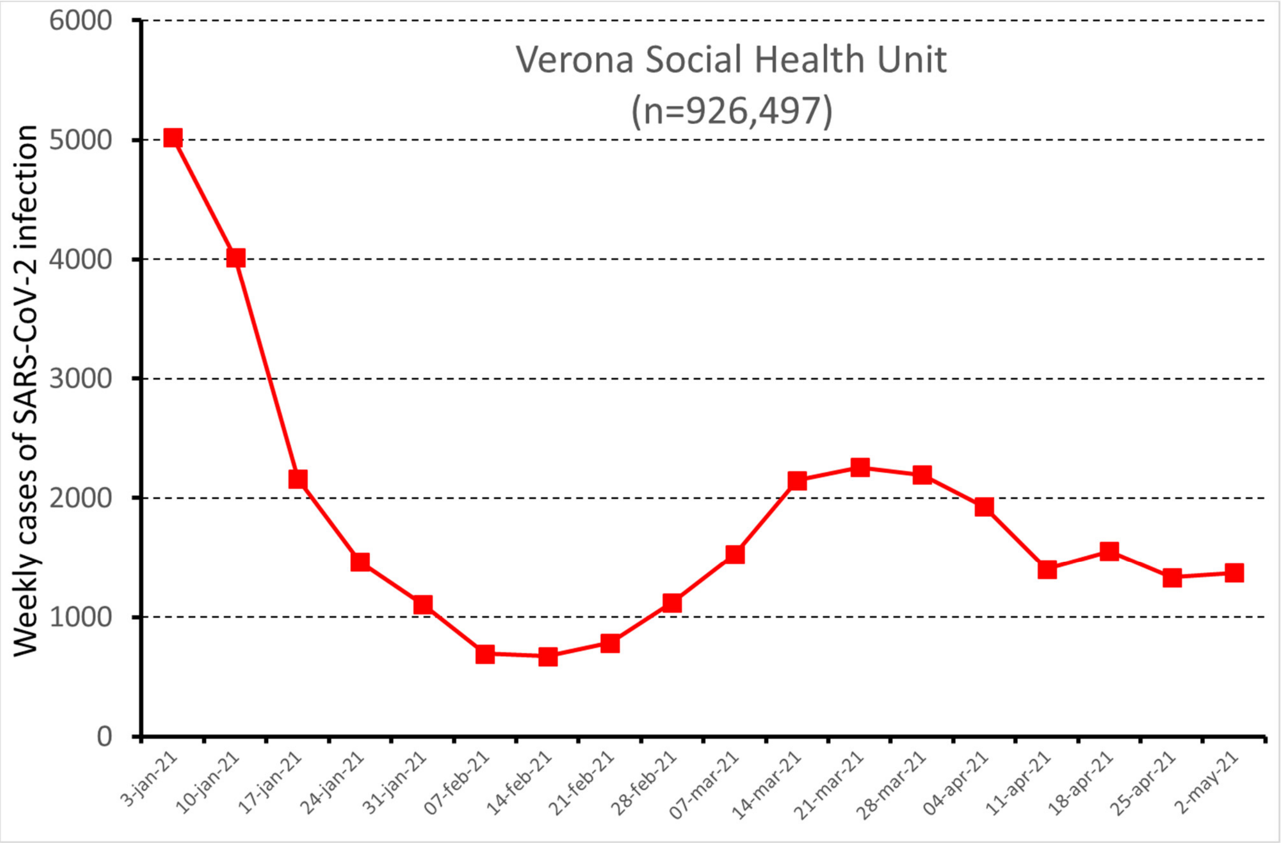

Figure S1: Weekly cases of SARS-CoV2 infection in the Verona Social Health Unit. The end of the week considered is reported on the X axis. The incidence of SARS-CoV-2 infection in Verona Social Health Unit (the local articulation of the public health authority system) was not stable, particularly in the first half of the observation period, when the number of weekly cases ranged from 5022 to 671. The incidence remained a bit more stable in March-April, when weekly cases ranged from 1333 to 2259.
